# Clinician Perspectives on the Extracorporeal Membrane Oxygenation Decision-Making Process

**DOI:** 10.1001/jamanetworkopen.2026.2044

**Published:** 2026-03-22

**Authors:** Derek R. Soled, Jacqueline M. Kruser, Alexander E. Jacobs, Rebecca M. Baron, Eddy Fan, James C. Henderson, Jonah Rubin

**Affiliations:** 1 Department of Internal Medicine-Pediatrics, Brigham and Women’s Hospital, Boston, Massachusetts; 2 Department of Internal Medicine-Pediatrics, Boston Children’s Hospital, Boston, Massachusetts; 3 Division of Allergy, Pulmonary, and Critical Care Medicine, University of Wisconsin, Madison; 4 Department of Medicine, Massachusetts General Hospital, Boston; 5 Department of Pulmonary and Critical Care Medicine, Brigham and Women’s Hospital, Boston, Massachusetts; 6 Interdepartmental Division of Critical Care Medicine, University of Toronto, Toronto, Ontario, Canada; 7 Institute of Health Policy, Management and Evaluation, University of Toronto, Toronto, Ontario, Canada; 8 Johns Hopkins University School of Medicine, Baltimore, Maryland; 9 Department of Pulmonary and Critical Care Medicine, Massachusetts General Hospital, Boston; 10 Corrigan Minehan Heart Center Intensive Care Unit, Massachusetts General Hospital, Boston

## Abstract

**Importance:**

Venovenous extracorporeal membrane oxygenation (VV ECMO) is a resource-intensive, life-sustaining technology to support patients with severe refractory respiratory failure. Its precise indications and contraindications are not standardized, and expert opinions are frequently changing, leading to variation in why and to whom VV ECMO is offered.

**Objective:**

To characterize the ways clinicians approach candidacy selection, the criteria considered, and the relative weight given to such variables.

**Design, Setting, and Participants:**

This qualitative study conducted virtual semistructured interviews of clinicians between September and December 2024. Transcripts were qualitatively analyzed from January to June 2025 using both inductive and deductive approaches to thematic analysis and line-by-line consensus coding. Participants were physicians and ECMO coordinators from various urban, rural, public, and private medical centers in 9 countries.

**Main Outcomes and Measures:**

Themes and subthemes that reflected clinicians’ experiences and views.

**Results:**

A purposeful sample of 45 clinicians directly involved in ECMO candidacy selection were contacted, of whom 24 (19 males [79%]) enrolled. Among these 24 participants interviewed, 21 (88%) were physicians and 3 (12%) were ECMO center coordinators; 8 (33%) practiced outside of the US. Five main themes were identified: (1) clinicians vary in their interpretation and incorporation of patient age, body mass index, and time on mechanical ventilation when selecting VV ECMO candidates; (2) perceived contraindications to VV ECMO are often flexible depending on various ethical and social criteria; (3) cognitive biases and heuristics affect the VV ECMO decision-making process; (4) institutional and cultural contexts shape individual VV ECMO candidacy decisions; and (5) participants provided suggestions to improve consistency in VV ECMO candidacy selection.

**Conclusions and Relevance:**

In this qualitative study, decisions to pursue VV ECMO for patients with severe respiratory failure were largely based on clinical judgments of suitability rather than objective guidelines. Variability in candidacy decision-making may lead to inconsistent or inequitable allocation.

## Key Points

**Question:**

In patients for whom venovenous extracorporeal membrane oxygenation (VV ECMO) may be medically indicated, how do clinicians approach candidacy determinations and consider different variables in the decision-making process?

**Findings:**

In this qualitative study of 24 physicians and ECMO coordinators from 9 countries, decisions to pursue VV ECMO for patients were largely based on clinical judgments of suitability rather than objective guidelines. Determining patient candidacy may be based on flexible interpretations of patient characteristics, biases, and social contexts.

**Meaning:**

The findings showed that, as ECMO is better understood and adopted in critical care medicine, ethical questions remain about how VV ECMO candidacy is variably determined.

## Teaser

This qualitative study explores the specific variables considered in selecting patients for venovenous extracorporeal membrane oxygenation (ECMO).

## Introduction

Venovenous extracorporeal membrane oxygenation (VV ECMO) is a resource-intensive, life-sustaining technology that supports patients with severe refractory respiratory failure. However, expert opinions delineating indications and contraindications to VV ECMO are frequently changing and have become less definitive over time.^1^ Professional society guidelines typically articulate candidacy considerations rather than hard criteria, and outcome prediction tools (eg, RESP [Respiratory ECMO Survival Prediction], SAVE [Survival After Venoarterial ECMO], PRESERVE [Predicting Death for Severe Acute Respiratory Distress Syndrome on VV ECMO] scores) demonstrate variable performance across contexts.^2,3,4^ There are also notable distinctions in how these decisions are made at ECMO centers in different countries.^5^

Lacking broad consensus-based approaches to identify candidates for VV ECMO, clinicians frequently must perform individualized, patient-level risk-benefit assessments. However, pure reliance on clinician discretion also risks between-center, within-center, and even intraclinician inconsistency,^5,6,7^ potentially leading to inefficient and inequitable allocation of this scarce resource. Factors as varied as patient race, sex, insurance status, and neighborhood have been associated with lower rates of ECMO utilization,^8^ and some studies^5,9^ (including unpublished work by D.R.S., 2026) have shown inconsistencies and biases in the ECMO allocation process. Despite concern for variability in patient selection and outcomes, there is little understanding of the mechanisms that lead to this variability, which requires an in-depth understanding of how ECMO candidacy decisions unfold in practice. This study aimed to characterize the ways clinicians approach candidacy selection, the criteria considered, and the relative weight given to such variables.

## Methods

### Design, Setting, and Participants 

We used a qualitative phenomenological research design^10,11,12,13^ to explore clinicians’ experiences with VV ECMO candidacy selection. The Mass General Brigham Institutional Review Board approved this qualitative study. All participating clinicians provided verbal informed consent. We followed the Consolidated Criteria for Reporting Qualitative Research (COREQ) reporting guideline.^14^

This study was conducted virtually with individuals from various medical centers in 9 countries. Among the clinicians who participated in a large, international survey of institutional ECMO candidacy decision-making processes, we purposefully selected individuals to interview to achieve broad experiential, geographic, and institutional (volume and protocol) diversity.^5^ Individuals who enrolled received a $50 Amazon.com gift card.

### Data Collection 

Sixty-minute interviews were conducted in English (by D.R.S.) between September and December 2024 using a semistructured interview guide (eAppendix in Supplement 1). The interviewer was blinded to the participants’ institutions and survey responses prior to interviews. Informed consent was verbally obtained to start each interview. Participants self-reported their clinical role, sex, race and ethnicity (Asian, Black, Hispanic, Middle Eastern, White, multiethnic, and other [other or not specified]), practice setting, and experience. Race and ethnicity data were collected to ensure heterogeneity. Interviews were recorded, transcribed (using TranscribeMe), and deidentified.

### Data Analysis

From January to June 2025, 2 of us (D.R.S. and J.R.) performed thematic coding and analysis^15,16^ using Dedoose, version 9.0.107 (SocioCultural Research Consultants).^17^ Both coders are physicians, and 1 physician (J.R.) had ECMO training. Each physician coded an initial set of 10 transcripts independently and blindly. After each transcript, they came together to discuss their codes, build a codebook (which was continually updated), and generate meaningful themes and subthemes. Thematic saturation was achieved by interview number 15. All interviews were analyzed for deeper characterization.

## Results

Of the 45 clinicians selected for interviews, 24 (53%) agreed to participate. The 24 interviewees consisted of 21 physicians (88%) and 3 ECMO coordinators (12%), of whom 19 (79%) were males and 5 (21%) were females; 8 clinicians (33%) practiced outside of the US (Table 1).

**Table 1.** Participant Characteristics

Characteristic	Participants, No. (%)
Role	
Physician	21 (88)
ECMO coordinator or APP	3 (12)
Sex	
Male	19 (79)
Female	5 (21)
Race and ethnicity^a^	
Asian	4 (17)
Black	2 (8)
Hispanic	0
Middle Eastern	2 (8)
White	15 (63)
Multiethnic	1 (4)
Other^b^	0
No. of y in ECMO candidacy decisions	
0-4	5 (21)
5-9	7 (29)
10-14	7 (29)
15-19	2 (8)
20-24	1 (4)
≥25	2 (8)
Country of practice	
Australia	1 (4)
Canada	1 (4)
Czech Republic	1 (4)
Germany	1 (4)
the Netherlands	1 (4)
Serbia	1 (4)
South Africa	1 (4)
UK	1 (4)
US	16 (67)
Hospital type	
Rural	2 (8)
Urban	22 (92)
Hospital funding	
Private	16 (67)
Public	8 (33)
Approximate No. of VV ECMO cases per y	
0-9	0
10-19	6 (25)
20-29	5 (21)
30-39	2 (8)
40-49	2 (8)
50-59	3 (13)
60-69	4 (17)
70-79	0
≥80	2 (8)

Abbreviations: APP, advanced practice provider; ECMO, extracorporeal membrane oxygenation; VV, venovenous.

^a^ Self-reported.

^b^ Includes individuals who identified as other race or ethnicity not otherwise specified.

Five themes were identified from the interviews: (1) clinicians vary in their interpretation and incorporation of patient age, body mass index (BMI), and time on mechanical ventilation when selecting VV ECMO candidates; (2) perceived contraindications to VV ECMO are often flexible depending on various ethical and social criteria; (3) cognitive biases and heuristics affect the VV ECMO decision-making process; (4) institutional and cultural contexts shape individual VV ECMO candidacy decisions; and (5) participants provided suggestions to improve consistency in VV ECMO candidacy selection. Representative quotes^18^ for themes 1 to 4 and subthemes are presented in Table 2 and Table 3, and participant suggestions are elaborated on in Table 4.

**Table 2.** Themes 1 and 2 With Representative Quotations

Themes and subthemes	Quotations
Theme 1: Clinicians vary in their interpretation and incorporation of patient age, BMI, and time on mechanical ventilation when selecting VV ECMO candidates	
Age	“Most of us in our group feel that chronologic age is not the same as physiologic age. And there’s a concept of frailty or frailty index. We have individuals who are markedly lower in age than 70 who are quite frail. And we have individuals in their 70s, or even early 80s, who are quite robust. And so, we tend to have that as a soft contraindication.”
	“We know that patients, even [those] who look great, who don’t have any diagnosed conditions in a normal set of labs, if they’re 85 years old, are not going to tolerate a physiologic insult as much as others, but, again, it’s relative. There are some patients who look great after a certain age. It probably is largely just a number, and so that’s why we don’t have it as an absolute contraindication.”
	“We try to get down and try to understand what the functional age is. So, a 72-year-old who’s been playing golf last Saturday might be a different 72-year-old who’s been in and out of the hospital. But I think more than anything else, we just use age both for young patients and older patients within the context of everything else happening. There are data to suggest that as you get older, ECMO may benefit you less and you may have a higher risk of mortality.”
	“I would say probably age would probably be the one that would get the most weight just because you’re going [to] be able to tell, you know, what’s going to be long-term goals for this patient and how resilient are they going to be.”
	“I feel like after a certain age, if you’re going to put somebody on ECMO, you need to do it early or not at all… just because they don’t tolerate these high [ventilatory] settings. Even if you don’t see anything objective in here, they accumulate this damage a lot worse at that age, I think.”
	“80 is probably beyond the extreme…we feel comfortable using age here as some sort of proxy for ability to rehab or to wean from the ventilator, things like that.”
BMI and technical access	“We don’t have a cutoff for BMI. It is an important factor to think about if you’re planning on actually cannulating the patient, obviously, in terms of technical difficulty. But we’ve had success with very high BMI patients.”
	“If we can lay eyeballs on the person, we can because if someone is built, you know, like they’re really stout or they’re almost built like a tree trunk or something, we tend to look at those [as] more favorable than someone who is all belly because those all-belly people have a difficult time flowing and they tend to be more unhealthy.”
	“If we cannot put a cannula in because you’re so obese, then that’s the cutoff.… [I]f you can cannulate, then it doesn’t matter what the BMI number is.”
	“Most of the data around obesity and ECMO are associated with problems with cannulation, right? So, we’ve got a [6-foot-5-inches] person with a BMI of 42, like a big muscular guy, we will consider putting them on, right?”
	“What we’ve realized is that around that BMI of 50 is where our flow rates become a huge problem for us. Like, you know, we’re just not able to, match their requirements in terms of like the flows that they require if you’re above that range.”
Ventilator time as proxy	“If we use ECMO upfront to prevent all this injury, that’s not salvage. That’s an active treatment.… If you use it as a salvage, once the ventilator has failed at the end of a week or two of treatment, most likely any benefit you could have had is gone and missed.”
	“There’s a feeling that the patients who do better are those who have an acute deterioration rather than those who’ve had a more incipient course to their illness. I think that’s where the time on the ventilator of 7 to 10 days [is important]… I’m less worried as an absolute like he’s been ventilated two weeks if it was still a reversible condition.”
	“We do not have strict rules about days. Majority of them were in the first few days. But if we ventilate this patient and we know he was ventilated in [a] protective manner all the time, we’ll place him on ECMO even on day 15. I don’t care if it’s really something that is completely reversible [rather than] just delay his process of dying, no.”
	“If there was a separate event… that timeline goes out the window. [Such as] let’s say on [the] 10th day, he developed ventilator-associated pneumonia with severe ARDS. And again, we placed him on ECMO, and he survived and went home without any consequences at the end.”
	“We really sort of treat that one as an absolute cutoff at about a week.… If that setting includes 100% oxygen for that whole time… we just assume their lungs to be pretty much fried. The dangers of high oxygen and fibrosis have been understated by a lot of the literature.”
Theme 2: Perceived contraindications to VV ECMO are often flexible depending on various ethical and social criteria	
Quality of life and functional outcome	“We place little weight on the absolute age and much more on the physiologic age or the frailty index.… You can walk into the room, is there family support there?”
	“So clinical frailty score is very important for us. If she’s attached to the bed and depending on someone, then definitely no. If she has a reasonable quality of life, okay.”
	“We declined ECMO because she had a combination of a too high BMI and was placed in a long-term facility and ambulating only with an [aid].”
	“If the patient is doing very poorly but is on no pressors, has normal kidney function, and looks generally fit… the gestalt is going to tell you that, ‘Oh, no, this patient is going to do well.’”
	“Catastrophic multi-organ failure in someone already with low function.… [ECMO’s] not going to save them…we usually will just say no.”
	“There’s also a value-based approach.… We think there’s more quality adjusted life years to be saved in younger patients.”
	“We do see as every 10 years beyond the age of 50, when you put them on ECMO, their…chance of a meaningful survival does start going down.”
Social support and post-ECMO disposition	“We kind of weighed those pros and cons of he is unhoused, so disposition could be a concern.… We did find a good medical power of attorney, a brother and a sister that were local that we could consult.”
	“It’s more the rest of the picture. Like does the patient have a social network? What is their general status? Do they go to their follow-up appointments?”
	“Is there family support there? We really need patient and family buy-in to get them through these acute illnesses let alone a transplant.”
Patient wishes and autonomy	“The patient was DNR and had written ‘I don’t want to be dependent on life support’.… Given his expressed wishes, ECMO wasn’t really within those goals.”
	“I do think his code status weighed in, but not because of the label itself, more that he was able to communicate, ‘I don’t want to be dependent on life support.’”
	“Individuals who lack independent living [or were] previously DNR or DNI [are] a herald sign that maybe they’re not in it for the long [haul].”
Moral distress and exceptions	“You cannot leave someone who is 25 or 26 years old, even with comorbidities…he’s too young not to try. We would feel very bad if we didn’t.”
	“People feel comfortable to use [a week cutoff] because it does ease the moral distress of it.… There is moral distress coming to the clinicians making the decisions. And they do look for those easier-to-navigate exit ramps.”
	“We pushed the parameters because she was 18. It did not have a good outcome and reinforced what the data out there shows.”
	“We just feel emotionally differently if you’ve got, for example, an obstetric patient or a young mother. It’s all kind of tossed into this muddy picture.… We were mortified of the optics of it, and we dragged our feet.”
	“A lot of emotional decision-making is being done and we acknowledge that.… There was a very young patient that we would have put on but he was not a transplant candidate, so we were talked out of it.… That was a difficult one emotionally for the team.”

Abbreviations: ARDS, acute respiratory distress syndrome; BMI, body mass index; DNI, do not intubate; DNR, do not resuscitate; ECMO, extracorporeal membrane oxygenation; VV, venovenous.

**Table 3.** Themes 3 and 4 With Representative Quotations

**Themes and subthemes**	**Quotations**
Theme 3: Cognitive biases and heuristics affect the VV ECMO decision-making process	
Loss aversion bias	“In the past, we have cannulated someone who had been ventilated for 19 days. And we did it because she was 18. We knew we were going outside of the usual parameters, but we also didn’t want to give up on an 18-year-old.”
	“In my experience, when patients are really young like less than 40, we tend to ignore a lot of very concerning stuff just because they’re so young. I think it becomes more of an emotional choice.”
	“A lot of the surgeons will not put a patient on who’s older than 75.… The older [the patient is], the more of a slam dunk that it’s going to be successful that [the ECMO consultant] really has to be.”
Status quo bias	“There’s never a re-consult.… I think it’s generally accepted that whoever takes the consult initially is the one who makes that decision, and everybody else gets to respect that.”
	“So that to me is really and truly what I feel like is our biggest issue here is [the ECMO center has] given the authority for providers to say no, but not the authority to say yes.”
	“Under normal circumstances, this just involves a conversation based on a clinical gestalt, our medical expert opinion on whether or not those patients are candidates or not.”
	“The conversations [of the ECMO team] don’t have a strict format…there are some times, though, when we do have sort of consensus calls.”
Sunk cost bias	“I suspect that there is almost certainly a sunk cost fallacy that is tied very closely to the surgeons and the surgeons’ groups.”
	“There can be a bias if the patient is a surgical or procedural patient.… [S]ometimes I feel like the criteria and the candidacy can be swayed a little bit towards that yellow or green flag because of the time and the relationship and, you know, ability to have a patient who you’ve done a procedure on.”
	“And when you have a surgical patient, it’s a shared decision-making model whereby a lot of weight is placed within the surgeon’s hands.”
	“There are things that can be completely thrown out the window when you have a vested interest in those patients.”
Recency bias	“[ECMO providers] use either recency bias or, you know, single anecdote cases to justify a specific decision.”
	“I think we’re a little bit guilty at times of sort of the last patient… ‘the last patient did well, we’ll do that one again.’”
	“We decided otherwise because there were too many similarities with these first patients who didn’t make it.”
Familiarity bias	“I think the issue in our healthcare system is there’s an element of chance with where you end up and who your referring doctor is into the pathway. A patient was referred in by the private pulmonologist for us to make an assessment of whether he could transfer to us, we had to say yes.”
	“Knowledge of the referrers.… We know that there are some very good hospitals, and if they refer, we know they’re in trouble…there are some worse hospitals.… [W]e’re more likely to say yes and almost look on it as our duty to rescue the patient from the clinician.”
	“We had a patient referred from a local hospital where a few of our guys did their training and they accepted it. Two days later, we get a guy from [a different hospital], and he was a no, and I think that sort of inconsistency is where people get upset.”
	“I think there’s also a difference in whether or not the patient is in-house versus at another facility.”
Group dynamics biases	“We are very much a consensus-driven group. If the head of ICU says yes, then do we actually even have a discussion afterwards? How much of that is going along with peer pressure? I think one or two strong voices for yes bring the whole team on board.”
	“There is a significant amount of inconsistency often depending on the comfort level of the cardiac surgeon. That has everything to do with their level of risk aversion.”
	“Without medical input, those patients would tend to lean away. But when one of the pulmonary attendings can say, ‘Oh, no, this is actually very treatable,’ that sort of changed the decision.”
	“Whoever took the consult initially is the one who makes that decision. The clinician who received the case might present it, but no one asks questions.”
Theme 4: Institutional and cultural contexts shape individual VV ECMO candidacy decisions	
Institutional capacity and resource limitations	“These are huge resource utilizers. We could care for a bunch of other patients in the same timeframe.… We have to consider this.”
	“We developed standard criteria that [are] less about the patient’s conditions and more about the number of available beds and machines. When we reach surge criteria, we have to follow [these] criteria and the review board literally votes.”
	“It’s hard to reallocate existing ECMO patients. Once someone is on, it is virtually impossible to take them off in favor of another patient. We’ve fortunately never been in that kind of situation.”
	“We would look at our capacity needs as well. Are we running six patients and we have a post-op patient on their way?”
	“Budget comes into it. If you’ve been funded, does that change our decision-making? We will say no, but I think it does.”
Transplant availability and long-term exit	“If [the transplant center tells] us a hard no … and we think there is established fibrosis, then we wouldn’t go on to put them on ECMO.”
	“We don’t use it as a bridge to transplant. The donors are too few and far between.… VV ECMO is for recovery. It’s not as a bridge.”
	“Our bridge to transplant is almost completely separate from our bridge to recovery.… [T]he bridge to recovery decision should be independent of transplant candidacy.”
	“Most centers use ages as a cutoff for [ECMO] that is the same cutoff for whether they are going to be a candidate for transplant.”
	“Historically [VV ECMO has been] a bridge to recovery. These days approximately half are either a bridge to transplantation or primary graft dysfunction after transplantation.”
Team culture and multidisciplinary decision-making	“Decisions where we decline require two physicians to assent. It can be unilaterally performed but only in extremis and occasionally overnight a third physician [sometimes functions] as the deal-breaker.”
	“It’s always a team decision. We’re generally five making the decision…the four intensivists on duty plus the ECMO consultant for that week.”
	“We use a WhatsApp group to govern our ECMO resource…six people, of which three need to be in agreement, though it is usually a consensus-driven group.”
	“We set up a Teams meeting with the ICU, interventional cardiologist, and surgical team. We will all meet, discuss the patient, and make a [shared] determination.”
	“Our ECMO council is a group of pulmonary and critical care physicians. Cardiothoracic surgeons, cardiac anesthesiologists, and other consultants can weigh in, but they do not get to vote on candidacy.”
	“The review board is ad hoc with senior leadership. We literally vote…it’s an odd number intentionally.”

Abbreviations: ECMO, extracorporeal membrane oxygenation; ICU, intensive care unit; VV, venovenous.

**Table 4.** Theme 5: Suggestions to Improve Consistency in VV ECMO Candidacy Selection

Strategy	Examples
Clear, written, evidence-based guidelines	Creating checklists with relative contraindications for candidacy that allow for individualization of candidacy decisions Separating surge guidelines from routine allocation criteria as well as in-house vs outside hospital referrals Working with division leadership to clearly delineate how, if at all, structural factors (eg, funding, beds) should contribute to candidacy selection
Team composition in a way that minimizes potential biases	Having ECMO decision councils that are multiperson and multidisciplinary, even overnight Outlining clear guidelines for when or how the primary team and/or consultants (eg, cardiac surgeons) should be able to contribute Creating formalized guidelines regarding if or when consensus must be reached vs holding a majority vote to cannulize Prioritizing the creation of psychological safety where team members can feel comfortable debating candidacy decisions In implementing systems that empower all voices and encourage dissent, considering a designated naysayer whose job is to force contrary discussion
Transparency in decision-making	Scheduling regular case reviews and debriefs to assess decision-making processes Tracking outcomes for patients deemed and denied candidacy, and using that data to concretely inform future cases Having multidisciplinary conferences to connect previous cases with the most recent literature and guidelines Separating ECMO candidacy decisions from transplant candidacy

Abbreviation: ECMO, extracorporeal membrane oxygenation.

### Theme 1: Variability in Interpreting and Incorporating Patient Variables and Time on Mechanical Ventilation in Selecting VV ECMO Candidates

All participants used patient age, BMI, and time spent on mechanical ventilation as the 3 main quantitative variables of several criteria for determining a patient’s VV ECMO candidacy. These variables usually served as proxies for other considerations, but these considerations differed among respondents.

#### Age

Age was used by every participant as a relative or absolute contraindication, with cutoffs ranging from 60 to 80 years. Participants agreed that physiologic age was more important than actual age. As a clinician shared, “Functional age matters more than biological age. A 70-year-old golfer and a 70-year-old nursing home patient are not the same.” Participants cited numerous reasons why age was important. Most used age as a proxy for frailty (or resilience) and overall outcome. Several shared that overall outcome and mortality were worse, and lung transplant was not an option in older age groups; thus, clinicians must be sure that a disease process is reversible before considering VV ECMO. Others believed VV ECMO was less appropriate in older patients with fewer remaining quality-adjusted life-years and thus were less eligible (“We’ve cannulated healthy 82-year-olds, but we weigh it heavily compared to a sick 52-year-old who can have a long life left.”).

#### BMI

Body habitus was seen as more important than BMI. Most participants considered BMI as a technical consideration related to vascular access (“It’s about access. Can we reach the neck? Is the pannus covering the groin?”). Relatedly, many remarked that patients with a higher BMI may require more (and mechanically unachievable) blood flow and suffer skin breakdown, and they assumed these patients had worse gas exchange given their habitus. As a clinician commented, “two patients might have identical BMIs, but the [6-foot-5-inches] muscular guy with big vessels in a big frame is a better fit.” Many participants mentioned the obesity paradox—the idea that weight may be associated with better outcomes for VV ECMO—as a reason that higher BMI may help one’s candidacy.^19^ Other participants believed higher BMIs reflected poor overall health and rehabilitative potential.

#### Ventilator Time

Participants used 7 to 14 days of mechanical ventilation as relative and absolute cutoffs. Participants cited these numbers, drawn from the EOLIA (ECMO to Rescue Lung Injury in Severe Acute Respiratory Distress Syndrome) trial criteria,^20^ as proxies for lung recoverability, extent of fibrosis, and oxygen toxic effect, regardless of disease process (“Time on a vent is a one-size-fits-all kind of thing for these other factors impacting recoverability and disease reversibility.”). Some clinicians said that ventilator times also signaled overall outcome and ability to tolerate other procedures. Other clinicians were more flexible, explaining that “it’s more the ventilatory journey than the absolute day count.” A patient may have been on suboptimal settings for the full duration or suffered other acute injuries that would be considered separately.

### Theme 2: Perceived Contraindications to VV ECMO 

Age, BMI, and time on mechanical ventilation were used as contraindications that varied with clinical contexts. Participants acknowledged that their candidacy assessments were sometimes subjective and based on “emotional decision-making.”

#### Quality of Life and Functional Outcome

Participants shared that age, BMI, and time on ventilator were used to determine a patient’s quality of life and functional outcome. As a participant noted, “We don’t want to save someone just because we can. They need a shot at real quality of life.” Another remarked, “If we think they’ll spend the rest of their life in a nursing home, that weighs in.” Relatedly, poor neurologic status or multiorgan failure strongly outweighed factors such as young age and normal BMI (“Prolonged down time leading someone to be not neurologically intact or other organ failure…those are bigger things to use than age.”).

#### Social Support

Several participants considered social supports and post-ECMO disposition. As a clinician asked, “If they don’t have family or housing, who’s going to help them get back on their feet?” Regarding an unhoused patient, a clinician commented, “We take that into account—especially if rehab won’t take them after.” Other participants evaluated candidacy decisions based on whether VV ECMO could be a bridge to recovery vs lung transplant. If transplant was an option, participants placed greater emphasis on patients’ social situations, wanting to ensure good medical compliance and follow-up.

#### Patient Wishes and Autonomy

Most participants agreed that when patients clearly expressed not “want[ing] to be dependent on machines,” VV ECMO would not be offered. One participant shared, “Sometimes families push, but if the patient said no to extraordinary measures beforehand, we respect that” because it is challenging to determine how long someone might require VV ECMO and whether other life-supporting technologies would be needed long-term.

#### Moral Distress and Exceptions

Participants believed that flexibility in weighing relative contraindications was crucial because “there are cases that just feel different—like young parents or health workers.” Another participant noted, “We occasionally bend the rules when we think it’s the right thing to do—we’re human. We’ve had huge debates about what’s fair versus what feels right.” Most participants shared that flexibility helped with navigating moral distress (“We want to try harder for an obstetric patient with young kids.”) and making otherwise difficult candidacy decisions with “easier-to-navigate exit ramps” based on flexible criteria that could be variably invoked.

### Theme 3: Cognitive Biases and Heuristics and the VV ECMO Decision-Making Process

Participants acknowledged that candidacy decisions were affected by decision-making shortcuts (or heuristics) and cognitive biases, defined as decisions deviating from impartial judgment without normative implications. Cognitive biases were acknowledged by more than 50% of the participants in our semistructured interviews.

#### Loss Aversion Bias

Many participants noted the loss aversion factors in their decisions regarding younger or previously healthy patients (“We’re more aggressive with younger patients, even if they’re not perfect candidates.”). When there was seemingly more to lose by saying no, participants more readily overlooked relative contraindications.

#### Status Quo Bias

Participants tended to follow through on a colleague’s initial opinion on VV ECMO candidacy, with other team members respecting the decision on consensus calls. As a participant said, “There’s never a re-consult or another discussion—presenting a case to the [ECMO] group is more a formality. We’re going to follow what the presenting clinician recommends or give them the reassurance they’re looking for.”

#### Sunk Cost Bias

Several participants noted a sunk cost bias for patients already receiving extensive care, especially postoperative patients. One clinician shared, “Surgical patients get a leg up. We’ve already invested in them.” Another clinician noted, “There’s pressure to escalate [to ECMO] when various consultants have already done so much and things deteriorate.”

#### Recency Bias

Most participants acknowledged that they were affected by recent or similar cases. Experience helped “justify a specific decision,” and participants usually recalled recent or memorable cases without comparing specific details.

#### Familiarity Bias

Most participants favored patients who were already known by their health system. Someone commented, “Referring hospitals don’t always know what we look for, so we’re more skeptical”; another clinician said, “Patients from our own ICU get more benefit of the doubt.” A few participants noted that patients who were referred privately or those with prior relationships with pulmonary specialists and intensivists seemed to get different treatment.

#### Group Dynamics Biases

Group dynamics often play a role in candidacy decisions. A participant mentioned, “Sometimes it depends on who’s in the room. One strong voice can sway the group.” Another clinician noted, “It’s consensus, but certain attending [physician]s tend to dominate.” Decisions also changed based on which specialties were included and informal hierarchies, such as whether surgeons performing the cannulation would get to vote. Most participants shared that candidacy decisions were often guided by the clinician on call at any given time, as different clinicians and specialties have different thresholds and comfort levels.

### Theme 4: Role of Institutional and Cultural Contexts in Individual VV ECMO Candidacy Decisions

Institutional structures, including funding and resource limitations as well as cultures, were often cited as factors in candidacy decisions. Responses were similar among participants across different hospital type (urban or rural), hospital funding (private or public), and country of practice.

#### Institutional Capacity and Resource Limitations

Participants noted that capacity and funding had a role in candidacy decisions. One clinician shared, “We’re funded for approximately 20 circuits a year. That shapes who we say yes to.” Another clinician commented, “We slip at times into accepting some patients because we’ve been quiet.” Since centers only have a certain number of ECMO circuits and beds, triage is affected by the time of year and context. During surges, for instance, a participant noted “tightening of selection criteria. For example, relative age cutoffs go down to an age less than 50 [years].” Relatedly, outside hospital transfers required additional resources. A participant commented, “We’ve probably got a lower threshold for doing it for our own inpatients…for the retrieval patients, criteria are more black and white.”

#### Transplant Availability

Whether a center had a transplant program available and whether ECMO could be a bridge to a transplant were factors to consider in candidacy. One participant commented, “If you don’t have transplant as a backup, you’re more careful with fibrotic lung disease.” While the “bridge to recovery decision should be independent of transplant candidacy,” several participants believed a noncandidate for a transplant would never be offered VV ECMO given the lack of a backup destination.

#### Team Culture and Multidisciplinary Decision-Making

Some institutions required unanimous consensus, while others required a majority vote. Decision thresholds changed by the time of day (eg, a physician could make a unilateral decision overnight), the primary ECMO consultant, and the specialties represented on the ECMO team (eg, surgery, pulmonology, and cardiology). One participant noted, “It’s consensus, but depending who’s on [the team], decisions shift.” Another participant said, “It all depends on who answers the phone and which team they’re from.”

### Theme 5: Improving Consistency in VV ECMO Candidacy Selection

Every participant in the study acknowledged inappropriate variability in candidacy selection, and most explained there were ways to improve internal consistency. They suggested creating clear guidelines, formalizing ECMO team composition, and ensuring transparency in decision-making. These strategies are summarized in Table 4.

## Discussion

Despite increasing ECMO utilization,^21,22,23,24,25,26^ understanding of how clinicians make candidacy decisions is lacking. To our knowledge, this study was the first to explore in detail the approach individual clinicians use in VV ECMO candidacy decision-making, including their degree of reliance on existing clinical guidelines. We identified themes related to patient-level variables, social factors, and institutional features that may impact these high-stakes decisions. We also elicited conceptual links binding clinical judgment and local practice culture to the existing literature, highlighting the ways in which evidence for VV ECMO is deployed in clinical contexts. In an evolving, data-sparse field,^27,28^ flexibility in decision-making is necessary but deserves to be scrutinized. By mapping the contours of clinical decision-making, our study may inform the development of more transparent, consistent, and equitable methods of VV ECMO candidate selection.

Several studies have proposed ways to weigh relative contraindications to ECMO,^29,30,31,32^ but most of these suggestions are guides in times of crisis (eg, the COVID-19 pandemic). Similarly, tools such as RESP scores broadly help categorize patients,^33,34^ but they are usually based on patients already on ECMO and do not account for more granular patient-level considerations or unique institutional experiences. These tools do not guide how to weigh specific contraindications or other social and cultural factors that affect candidacy decisions. From an ethics perspective, most ethics research on ECMO relates to allocation and principles of justice, such as whether older patients should have the same access to life-sustaining therapies as younger patients (also known as the fair innings principle^35^), as opposed to the ethics of decision-making with limited data and flexibility. A potentially better and more equitable framework for candidacy decisions may be domain-based decision-making, in which clinicians identify the specific, overarching destination-related reasons (domains, as opposed to contraindications) that a patient cannot get ECMO, using specific data points to support the domain rather than independent contraindications.^36^ Strategies suggested by participants (Table 4) can also be adopted to create frameworks for consistency, transparency, and equity in the decision-making process.

Some themes that we identified from the interviews were at times contradictory. For instance, the same group dynamics and cognitive biases may both raise or lower the threshold for candidacy, likely due to variability in the VV ECMO goal (recovery vs transplant) that could not always be parsed out in interviews. In addition, candidacy decisions may vary both between and within centers. The fact that, at times, certain themes were contradictory further demonstrates the many differences in how centers approach selection. Research on ECMO is constantly evolving, and centers are updating their processes in real time; our interviews represent participants’ views at one point in time. Participants were generally supportive of some flexibility in selection because so much is still unknown about ECMO.^37^ Our study reaffirms that candidacy decision-making is an extremely nuanced and subjective process, which is both necessary and can exacerbate health inequities,^8,9^ an issue that is well-recognized in critical care.^38,39^

There is modest empiric research to date on physicians’ perspectives and experiences with ECMO decision-making. Most of the research focuses on patient and family perspectives on ECMO decisions,^40,41,42^ and those that do focus on clinicians are usually hypothetical or examine the decision itself^43^ as opposed to the decision-making process. While our study did not involve nonclinical participants, better understanding the selection process and how it affects patients is paramount to improving resource allocation and strengthening physician-patient relationships.

Our study has important implications. Given the lack of standardization in approaches to candidacy selection, critical care units could benefit from internal reviews for consistency and cross-center collaboration, not only for VV ECMO but also for venoarterial ECMO and other forms of mechanical circulatory support, including left ventricular assist devices. We hypothesized that, similar to ECMO, resource distribution in critical illness is dependent on structural, interpersonal, and individual factors.^29,44,45,46,47^ Further research is needed on the intersection of clinical ethics and medicine in different settings.

### Limitations

This study has several limitations. First, this sample was small, overly represented by the US (and other countries in the Global North), and purposely selected from a cohort of survey respondents, which may have led to sampling bias. Second, participants may not precisely represent what happens in practice and may have difficulty reflecting on their own biases (eg, recalling only memorable experiences). Third, we sought only perspectives on VV ECMO candidacy selection, and findings may not apply to other forms of ECMO.

## Conclusions

Decisions to pursue VV ECMO for patients with severe respiratory failure were largely based on clinical judgments of suitability rather than objective guidelines. Our findings suggest that determining a patient’s candidacy may be based on different and flexible interpretations of patient characteristics, social contexts, and biases. Variability in VV ECMO candidacy decision-making may lead to inconsistent allocation, and further research is needed to identify more equitable strategies.

## Notes

Supplement 1. **eAppendix.** Semi-Structured Interview Guide

Supplement 2.
Data Sharing Statement

## References

[1] Rubin J, Fan E. The need and approach for critical assessment of extracorporeal membrane oxygenation candidacy decision-making: a call to action. Chest. 2023;164(2):299-301. doi:10.1016/j.chest.2023.02.025 37558328

[2] Kang HR, Kim DJ, Lee J, . A comparative analysis of survival prediction using PRESERVE and RESP scores. Ann Thorac Surg. 2017;104(3):797-803. doi:10.1016/j.athoracsur.2017.01.052 28410641

[3] Amin F, Lombardi J, Alhussein M, . Predicting survival after VA-ECMO for refractory cardiogenic shock: validating the SAVE Score. CJC Open. 2020;3(1):71-81. doi:10.1016/j.cjco.2020.09.011 33458635 PMC7801193

[4] Moyon Q, Pineton de Chambrun M, Lebreton G, Chaieb H, Combes A, Schmidt M. Validation of survival prediction models for ECMO in Sars-CoV-2-related acute respiratory distress syndrome. Crit Care. 2022;26(1):187. doi:10.1186/s13054-022-04039-4 35729619 PMC9210051

[5] Henderson JC, Ilg AM, Meeker MA, . Variability in venovenous extracorporeal membrane oxygenation candidacy decision-making: an international survey. Crit Care Med. 2025;53(12):e2440-e2451. doi:10.1097/CCM.0000000000006889 41090991

[6] Rubin J, Alves BR, Padrao EMH, . Candidacy decision making for extracorporeal cardiopulmonary resuscitation (ECPR): lessons from a single-center retrospective analysis. J Cardiothorac Vasc Anesth. 2025;39(10):2606-2614. doi:10.1053/j.jvca.2025.04.031 40374393

[7] Rubin J, Witkin AS, Crowley JC, . Venovenous extracorporeal membrane oxygenation candidacy decision-making: lessons and hypotheses from a single-center observational analysis. Chest. 2024;166(3):491-501. doi:10.1016/j.chest.2024.02.042 38423278

[8] Mehta AB, Taylor JK, Day G, Lane TC, Douglas IS. Disparities in adult patient selection for extracorporeal membrane oxygenation in the United States: a population-level study. Ann Am Thorac Soc. 2023;20(8):1166-1174. doi:10.1513/AnnalsATS.202212-1029OC 37021958 PMC10405618

[9] Jacobs AE, Soled DR, Rubin J. Judgment under uncertainty: a case-based analysis of cognitive bias in ECMO candidacy decision-making. Chest. 2026:S0012-3692(26)00136-4. doi:10.1016/j.chest.2026.01.014 41638359

[10] Bradshaw C, Atkinson S, Doody O. Employing a qualitative description approach in health care research. Glob Qual Nurs Res. 2017;4:2333393617742282. doi:10.1177/2333393617742282 29204457 PMC5703087

[11] van Manen M. But is it phenomenology? Qual Health Res. 2017;27(6):775-779. doi:10.1177/1049732317699570 28682717

[12] Connelly LM. What is phenomenology? Medsurg Nurs. 2010;19(2):127-128. 20476524

[13] Sundler AJ, Lindberg E, Nilsson C, Palmér L. Qualitative thematic analysis based on descriptive phenomenology. Nurs Open. 2019;6(3):733-739. doi:10.1002/nop2.275 31367394 PMC6650661

[14] Tong A, Sainsbury P, Craig J. Consolidated criteria for reporting qualitative research (COREQ): a 32-item checklist for interviews and focus groups. Int J Qual Health Care. 2007;19(6):349-357. doi:10.1093/intqhc/mzm042 17872937

[15] Corbin J, Strauss A. Basics of Qualitative Research, 3rd ed: Techniques and Procedures for Developing Grounded Theory. SAGE Publications, Inc; 2008.

[16] Patton MQ. Qualitative Research & Evaluation Methods. 4th ed. SAGE Publications, Inc; 2015.

[17] Dedoose version 9.0.107, cloud application for managing, analyzing, and presenting qualitative and mixed method research data. 2023. Accessed September 28, 2025. http://www.dedoose.com

[18] Lingard L. Beyond the default colon: effective use of quotes in qualitative research. Perspect Med Educ. 2019;8(6):360-364. doi:10.1007/S40037-019-00550-7 31758490 PMC6904374

[19] Ripoll JG, ElSaban M, Nabzdyk CS, . Obesity and extracorporeal membrane oxygenation (ECMO): analysis of outcomes. J Cardiothorac Vasc Anesth. 2024;38(1):285-298. doi:10.1053/j.jvca.2023.10.025 37953169

[20] Combes A, Hajage D, Capellier G, ; EOLIA Trial Group, REVA, and ECMONet. Extracorporeal membrane oxygenation for severe acute respiratory distress syndrome. N Engl J Med. 2018;378(21):1965-1975. doi:10.1056/NEJMoa1800385 29791822

[21] Saxena A, Curran J, Ahmad D, . Utilization and outcomes of V-AV ECMO: a systematic review and meta-analysis. Artif Organs. 2023;47(10):1559-1566. doi:10.1111/aor.14610 37537953

[22] Papanikolaou V, Goh E, Carrandi A, . Health resource utilization and outcomes among patients who receive extracorporeal membrane oxygenation. CJC Open. 2025;7(6):750-758. doi:10.1016/j.cjco.2025.03.019 40586013 PMC12198622

[23] McCarthy FH, McDermott KM, Kini V, . Trends in U.S. extracorporeal membrane oxygenation use and outcomes: 2002-2012. Semin Thorac Cardiovasc Surg. 2015;27(2):81-88. doi:10.1053/j.semtcvs.2015.07.005 26686427 PMC4780346

[24] Sauer CM, Yuh DD, Bonde P. Extracorporeal membrane oxygenation use has increased by 433% in adults in the United States from 2006 to 2011. ASAIO J. 2015;61(1):31-36. doi:10.1097/MAT.0000000000000160 25303799

[25] Stentz MJ, Kelley ME, Jabaley CS, . Trends in extracorporeal membrane oxygenation growth in the United States, 2011-2014. ASAIO J. 2019;65(7):712-717. doi:10.1097/MAT.0000000000000872 30247176

[26] Sanaiha Y, Bailey K, Downey P, . Trends in mortality and resource utilization for extracorporeal membrane oxygenation in the United States: 2008-2014. Surgery. 2019;165(2):381-388. doi:10.1016/j.surg.2018.08.012 30253872

[27] Feldhaus D, Brodie D, Lemaitre P, Sonett J, Agerstrand C. The evolution of the use of extracorporeal membrane oxygenation in respiratory failure. Membranes (Basel). 2021;11(7):491. doi:10.3390/membranes11070491 34208906 PMC8305045

[28] Brodie D. The evolution of extracorporeal membrane oxygenation for adult respiratory failure. Ann Am Thorac Soc. 2018;15(suppl 1):S57-S60. doi:10.1513/AnnalsATS.201705-386KV 29461889

[29] Ehmann MR, Zink EK, Levin AB, . Operational recommendations for scarce resource allocation in a public health crisis. Chest. 2021;159(3):1076-1083. doi:10.1016/j.chest.2020.09.246 32991873 PMC7521357

[30] Prekker ME, Brunsvold ME, Bohman JK, . Regional planning for extracorporeal membrane oxygenation allocation during Coronavirus Disease 2019. Chest. 2020;158(2):603-607. doi:10.1016/j.chest.2020.04.026 32339510 PMC7182515

[31] Murugappan KR, Walsh DP, Mittel A, Sontag D, Shaefi S. Veno-venous extracorporeal membrane oxygenation allocation in the COVID-19 pandemic. J Crit Care. 2021;61:221-226. doi:10.1016/j.jcrc.2020.11.004 33220575 PMC7664357

[32] Dao B, Savulescu J, Suen JY, Fraser JF, Wilkinson DJC. Ethical factors determining ECMO allocation during the COVID-19 pandemic. BMC Med Ethics. 2021;22(1):70. doi:10.1186/s12910-021-00638-y 34074282 PMC8169422

[33] Majithia-Beet G, Naemi R, Issitt R. Efficacy of outcome prediction of the respiratory ECMO survival prediction score and the predicting death for severe ARDS on VV-ECMO score for patients with acute respiratory distress syndrome on extracorporeal membrane oxygenation. Perfusion. 2023;38(7):1340-1348. doi:10.1177/02676591221115267 35830605

[34] Giordano L, Francavilla A, Bottio T, . Predictive models in extracorporeal membrane oxygenation (ECMO): a systematic review. Syst Rev. 2023;12(1):44. doi:10.1186/s13643-023-02211-7 36918967 PMC10015918

[35] Williams A. Intergenerational equity: an exploration of the ‘fair innings’ argument. Health Econ. 1997;6(2):117-132. doi:10.1002/(SICI)1099-1050(199703)6:2<117::AID-HEC256>3.0.CO;2-B 9158965

[36] Rubin J. Domain-based decision making, in ECMO and beyond: sometimes more is more. Am J Bioeth. 2025;25(8):35-37. doi:10.1080/15265161.2025.2525766 40736982

[37] Soled DR. Structured autonomy: increasing self-governance in modern medicine. J Clin Ethics. 2025;36(3):230-250. doi:10.1086/736144 40789088

[38] McGowan SK, Sarigiannis KA, Fox SC, Gottlieb MA, Chen E. Racial disparities in ICU outcomes: a systematic review. Crit Care Med. 2022;50(1):1-20. doi:10.1097/CCM.0000000000005269 34636803

[39] Mitchell HK, Reddy A, Perry MA, Gathers CA, Fowler JC, Yehya N. Racial, ethnic, and socioeconomic disparities in paediatric critical care in the USA. Lancet Child Adolesc Health. 2021;5(10):739-750. doi:10.1016/S2352-4642(21)00161-9 34370979

[40] Yang X, Lin Y, Tang A, . Tough choices: the experience of family members of critically ill patients participating in ECMO treatment decision-making: a descriptive qualitative study. BMC Med Inform Decis Mak. 2025;25(1):65. doi:10.1186/s12911-025-02876-1 39920721 PMC11806576

[41] Moynihan KM, Dorste A, Siegel BD, Rabinowitz EJ, McReynolds A, October TW. Decision-making, ethics, and end-of-life care in pediatric extracorporeal membrane oxygenation: a comprehensive narrative review. Pediatr Crit Care Med. 2021;22(9):806-812. doi:10.1097/PCC.0000000000002766 33989251

[42] Shi J, Dai J, Pan J, . Between hope and harm: a meta-synthesis of family decision-making agonies in ECMO initiation crossroads. Nurs Crit Care. 2025;30(6):e70204. doi:10.1111/nicc.70204 41074597

[43] Piscitello GM, Bermea RS, Stokes JW, . Clinician ethical perspectives on extracorporeal membrane oxygenation in practice. Am J Hosp Palliat Care. 2022;39(6):659-666. doi:10.1177/10499091211041079 34414798 PMC8858336

[44] Fraser KD, Estabrooks C. What factors influence case managers’ resource allocation decisions? a systematic review of the literature. Med Decis Making. 2008;28(3):394-410. doi:10.1177/0272989X07312709 18480042

[45] Emanuel EJ, Persad G, Upshur R, . Fair allocation of scarce medical resources in the time of Covid-19. N Engl J Med. 2020;382(21):2049-2055. doi:10.1056/NEJMsb2005114 32202722

[46] Emanuel EJ, Persad G. The shared ethical framework to allocate scarce medical resources: a lesson from COVID-19. Lancet. 2023;401(10391):1892-1902. doi:10.1016/S0140-6736(23)00812-7 37172603 PMC10168660

[47] Furnham A. Factors relating to the allocation of medical resources. J Soc Behav Pers. 1996;11(3):615-624. 11660693

