# Supplementary material for: Clinician Perspectives on the Extracorporeal Membrane Oxygenation Decision-Making Process
Source: JAMA Netw Open. 2026 Mar 22;9(3):e262044. doi: 10.1001/jamanetworkopen.2026.2044 (PMC13006854; doi:10.1001/jamanetworkopen.2026.2044)
Supplement: Supplement 1. — eAppendix. Semi-Structured Interview Guide [file jamanetwopen-e262044-s001.pdf]

# Supplemental Online Content

Soled DR, Kruser JM, Jacobs AE, et al. Clinician perspectives on the extracorporeal membrane oxygenation decision-making process. *JAMA Netw Open*. 2026;9(3):e262044. doi:10.1001/jamanetworkopen.2026.2044

**eAppendix.** Semi-Structured Interview Guide

This supplemental material has been provided by the authors to give readers additional information about their work.

© 2026 Soled DR et al. *JAMA Network Open*

**eAppendix. Semi-structured interview guide.**

**Participant ID: ________________ Site:________________**
**Date:________________**
**Pre-Interview checklist**

- □ Information Sheet emailed to participant
- □ Confirmed recording equipment present and working

Thank you for meeting with me and your willingness to participate. Your input is very valuable. This interview should take no longer than 60 minutes. Feel free to stop me at any time for any reason. Before we begin, do we have your consent to record this study interview, so that we make sure we have a complete and accurate record of our conversation?

**Questions** (Data desired listed as subpoints)

1. First, we would like to ask you several demographics and characteristics questions. Can you please tell me the following: Are you involved in extracorporeal membrane oxygenation (ECMO) candidacy decision-making as a physician or coordinator? What year did you start working in ECMO candidacy selection? What country do you work in? Do you work in an urban or rural medical center? Would you classify your center as public or private? Approximately how many cases of veno-venous (VV) ECMO does your medical center perform each year? What is your sex? Please identify your race and ethnicity as one of the following: Asian, Black, Hispanic, Middle Eastern, Multiethnic, White, or Other (defined as an other race or ethnicity not previously mentioned).

2. The focus of this interview is VV ECMO for respiratory failure, and we are interested in your thoughts and opinions about how and why decisions are made about whether a patient is or is not a candidate for VV ECMO. To start, please recall a recent patient at your hospital for whom VV ECMO was considered—this could be a person who ended up receiving ECMO or not. Could you tell me about this case, and how the decision about ECMO was made?

   - What do you think went well in the decision-making process for this case?
   - In what ways did the decision-making process fall short for this patient?
   - Do you think this case represents how the process usually goes at your hospital? [In what ways does it differ / in what ways does it represent the usual process?]

3. We recently conducted a large survey of ECMO centers to identify which criteria are used most as absolute and relative contraindications for candidacy. Three of the most common answers were age, BMI, and time on ventilator. I would like to ask you about each of these individually. First, how is age considered at your hospital, as a criterion for VV ECMO candidacy?

   - Would you consider a certain age to be an absolute contraindication for ECMO? What would the cutoff be?
   - Why do you believe age is an important factor in VV ECMO decision making?

© 2026 Soled DR et al. *JAMA Network Open*

4. Second, how do you consider BMI as a criterion for VV ECMO candidacy?

    - Would you consider a certain BMI as an absolute contraindication for ECMO? What would the cutoff be?
    - Why do you believe this is important for VV ECMO decision making?

5. Third, how do you consider the time the patient has already been receiving mechanical ventilation in ECMO decision making?

    - Would you consider a certain time as an absolute contraindication? What would that time be?
    - Why do you believe this variable is important?

6. Can you talk me through how you incorporate each of these three variables, together, in your decision-making process about VV ECMO candidacy?

    - How do you weigh these individual variables?
    - How do these individual variables (or others) compare with your overall clinical gestalt about whether a patient is a good candidate for VV ECMO? What do you think informs your clinical gestalt that isn't captured in these variables?
    - What if the patient has one contraindication, but otherwise meets the criteria?

7. To what extent do you believe the VV ECMO decision-making process is consistent across patients at your hospital?

    - Have you ever noticed inconsistencies where one patient is deemed a candidate whereas another with similar conditions and prognosis is not?
    - Have you yourself have been inconsistent? Are you willing to share an example?
    - How do you (personally) or the processes at your hospital seek to minimize these inconsistencies?
    - How do you (personally) or the processes at your hospital seek to minimize bias in deciding candidacy?

8. This concludes our interview. Do you have any other thoughts on ECMO candidacy, decision-making, inconsistencies, and biases in your hospital and practice?

[Participants were then asked to respond to one hypothetical scenario. Their responses were not used for this paper's analysis and thus have been omitted from the supplement.]

© 2026 Soled DR et al. *JAMA Network Open*
