# Supplementary material for: Clinician Perspectives on the Extracorporeal Membrane Oxygenation Decision-Making Process
Source: JAMA Netw Open. 2026 Mar 22;9(3):e262044. doi: 10.1001/jamanetworkopen.2026.2044 (PMC13006854; doi:10.1001/jamanetworkopen.2026.2044)
Supplement: Supplement 2. — Data Sharing Statement [file jamanetwopen-e262044-s002.pdf]

Data Sharing Statement

Soled. Clinician Perspectives on the Extracorporeal Membrane Oxygenation Decision-Making Process. *JAMA Netw Open*. Published March 20, 2026.
doi:10.1001/jamanetworkopen.2026.2044

Data
Data available: No

Additional Information
Explanation for why data not available: Given the nature of qualitative research, it is impossible to fully anonymize interview data. It is possible to identify participants from their responses even after redacting names, institutions, and other identifying information. Thus, we are not able to share the interview transcripts and protect the privacy of research participants. If there are other researchers interested in collaborating on further research using this data set, please reach out to me (D.R.S.), and we can explore the potential to collaborate, which would include adding collaborators to the IRB in order to gain access to interview data.
